# Case Report: Early diaphragmatic plication for combined phrenic and recurrent laryngeal nerve injury after VATS thymectomy

**DOI:** 10.3389/fmed.2026.1834703

**Published:** 2026-06-30

**Authors:** Hai Li, Shi dan Li, Qi chang Jiang, Yang Liu, Gang Xu

**Affiliations:** 1 Department of Thoracic and Cardiovascular Surgery, The Second Affiliated Hospital of Zunyi Medical University, Zunyi, Guizhou, China; 2 Department of Critical Care Medicine, The Affiliated Hospital of Zunyi Medical University, Zunyi, Guizhou, China

**Keywords:** aerophagia, diaphragmatic plication, phrenic nerve injury, recurrent laryngeal nerve, respiratory failure, VATS thymectomy

## Abstract

Video-assisted thoracoscopic surgery (VATS) thymectomy may cause phrenic nerve injury (PNI) or recurrent laryngeal nerve (RLN) injury. Combined PNI and RLN injury is rare but can cause fatal respiratory failure. This study reported a case of VATS thymectomy in a 50-year-old man. The patient received a VATS thymectomy at an external hospital and presented to our hospital 10 days later with severe dyspnea and abdominal distention. Pre-surgery radiologic examination identified a 1.6-cm anterior mediastinal mass, and thymoma was considered. The pathology report confirmed benign reactive lymph node hyperplasia. The patient was hypoxemic with a PaO_2_ of 54 mmHg in room air. Pulmonary CT angiography ruled out pulmonary embolism. Laryngoscopy and fluoroscopic sniff test showed paradoxical left hemidiaphragm movement, leading to a diagnosis of left vocal cord paralysis. Chest CT revealed left lower lobe atelectasis with severe gastric distension that compressed the left hemithorax. Removal of the mucus plugs by bronchoscopy gave short-term relief. Bilevel positive airway pressure (BiPAP) caused a counterintuitive increase in gastric distension, which is expected after air enters an incompetent glottis. We proposed that combined PNI and RLN injury leads to ineffective cough, aerophagia, and a self-reinforcing pathophysiological cycle. Conservative treatment failed. Single-incision VATS left diaphragmatic plication was performed on hospital day 7 at our hospital. Oxygen saturation returned to normal, the lung re-expanded, and the abdominal girth reduced by 14 cm in less than 48 h. This report demonstrated the effect of combined PNI and RLN injury after VATS thymectomy and suggested early diaphragmatic plication as a potential therapeutic option in cases failing to respond to conservative treatment.

## Introduction

VATS thymectomy has become a popular procedure to resect thymic tumors and other mediastinal masses. It has a lower morbidity than median sternotomy in most cases (1). The thymus is located in close proximity to the phrenic nerve and RLN, which can be injured during perioperative dissection. Unilateral nerve injury is common and usually presents with symptoms including elevation of one-half of the diaphragm and hoarseness, which most patients can tolerate. In contrast, combined PNI and RLN injury is rare and can result in severe respiratory failure, especially in cases where secretions are not cleared effectively, and secondary gastrointestinal dilatation develops to further impede mechanics (2).

The surgical approach, dissection plane, and mediastinal manipulation for benign reactive lymph node hyperplasia and thymic tumors are identical. However, tumors adjacent to neurovascular structures are associated with a higher likelihood of nerve injury. This study reported a case of severe aerophagia complicated by combined PNI and RLN injury, which seemed to increase the severity of respiratory failure. Meanwhile, this study presented the result of early single-incision VATS diaphragmatic plication in this patient.

## Case presentation

A 50-year-old man underwent a VATS thymectomy at an external hospital for a 1.6-cm anterior mediastinal mass, which appeared suspicious for thymoma on radiologic examination. The patient had not undergone any significant medical treatment and denied a history of chronic lung disease or tobacco use. Based on the operative report, a three-port surgery on the left side of the body was performed: one camera port was positioned at the fifth intercostal space, and two working ports were placed at the third and sixth intercostal spaces, respectively. The pneumothorax was created by insufflating carbon dioxide to 8 mmHg. Mediastinal dissection was performed with an ultrasonic energy device. The mass with thymic tissue was dissected within 95 min, accompanied by minor bleeding. According to the medical record, the mass was attached to the left phrenic nerve and required separation by sharp dissection. Hoarseness developed post-surgery, and the patient was placed on voice rest. The patient was discharged on day 3 after surgery. The patient did not undergo preoperative pulmonary function tests, as such tests are not required in the absence of respiratory symptoms. Phone contact with the operating surgeon verified these operative details, but intraoperative images or videos were not retrieved.

Ten days post-surgery, the patient presented to the emergency department at our hospital with more severe dyspnea and abdominal distention. The patient had significant respiratory distress and orthopnea, and he was unable to lie supine. Vital signs were SpO_2_ 88% in room air, respiratory rate 28 breaths/min, heart rate 102 beats/min, blood pressure 134/82 mmHg, and temperature 36.6 degrees Celsius. Physical examination revealed hoarseness, decreased breath sounds in the left hemithorax, and an extremely large abdomen that was soft and non-tender without peritoneal signs. There was no jugular venous distension or cyanosis. The patient was transferred to an intensive care unit and monitored. Supportive treatment was provided. Enoxaparin (40 mg) was administered subcutaneously daily for prophylactic anticoagulation throughout the hospitalization.

**Figure 1:**
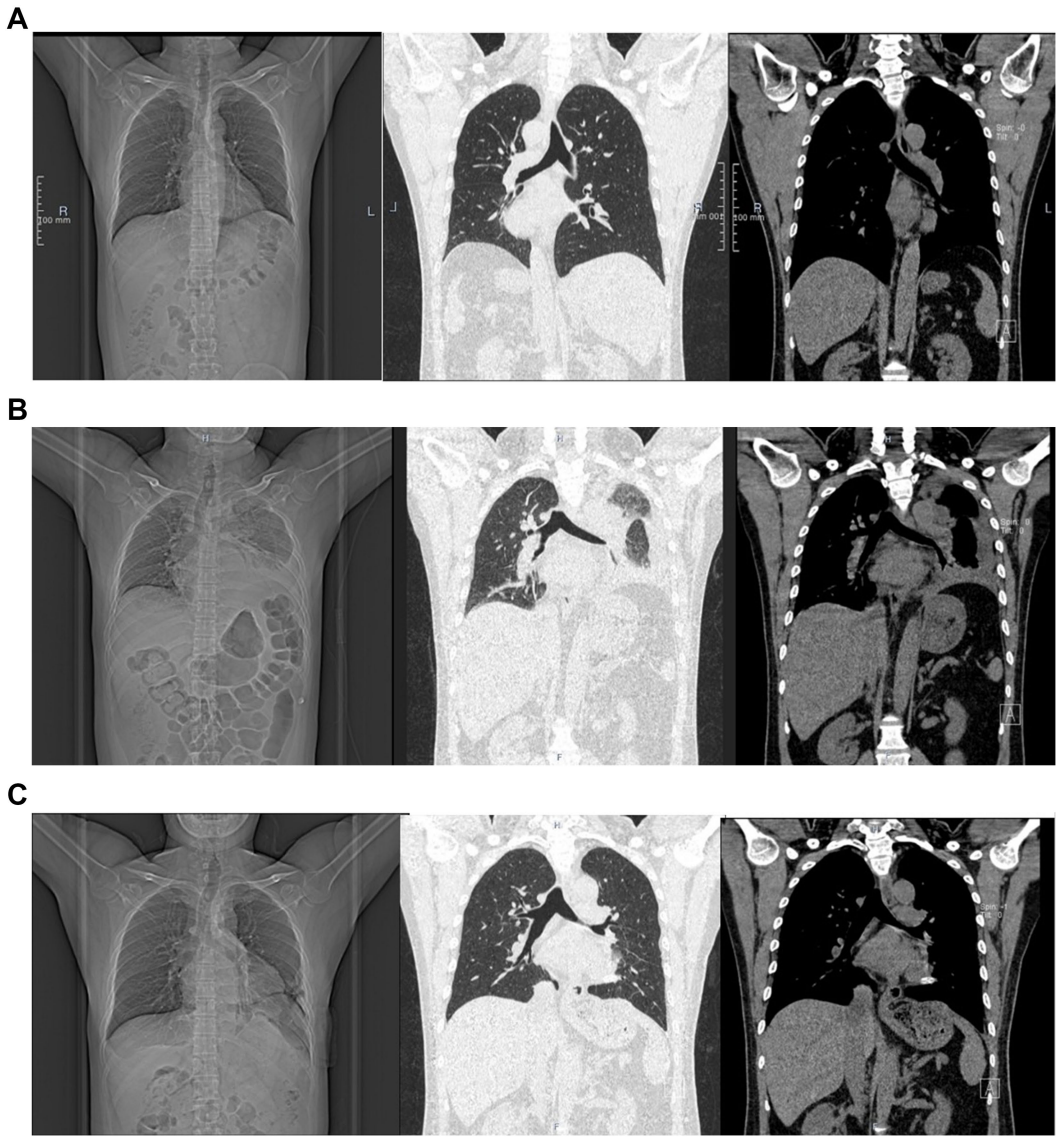
Radiological findings. **(A)** Preoperative chest CT obtained prior to thymectomy demonstrates normal bilateral diaphragmatic position, fully expanded lungs, and absence of gastric distension. **(B)** Post-thymectomy imaging at emergency presentation (postoperative day 10) demonstrates marked elevation of the left hemidiaphragm, complete left lower lobe atelectasis, and massive gastric distension compressing the left hemithorax; CT pulmonary angiography confirmed the absence of pulmonary embolism. **(C)** Chest CT obtained 48 h after single-incision VATS diaphragmatic plication demonstrating complete left lung re-expansion, restoration of diaphragmatic position at the level of the posterior eighth rib, and resolution of gastric distension.

Room air arterial blood gas analysis showed a pH of 7.46, PaCO_2_ of 32 mmHg, and PaO_2_ of 54 mmHg, suggesting hypoxemic respiratory failure with compensatory hyperventilation. Laboratory investigation revealed a white blood cell count of 8.2 * 10^9^ per liter with normal differential, a C-reactive protein of 18 mg per liter, and normal procalcitonin. Contrast-enhanced chest computed tomography (CT) with CT pulmonary angiography demonstrated a completely atelectatic left lower lobe, an elevated left hemidiaphragm, and severe gaseous distention of the stomach and colon compressing the left hemithorax (Figure 1B). Pulmonary embolism was ruled out. Compared with the preoperative images from the external hospital, these postoperative findings were new (Figure 1A).

Flexible laryngoscopy on hospital day 1 revealed that the left vocal cord was held in the para-median position with persistent glottic incompetence, consistent with left RLN injury. A bedside swallowing assessment was performed, and the patient was put on a soft diet with thickened liquids. Video fluoroscopic swallowing evaluation was not performed. There was no clinical confirmation of aspiration in the hospital. A fluoroscopic sniff test showed that the left hemidiaphragm was moved cephalad during rapid inspiration, leading to a diagnosis of left PNI. Bronchoscopy found open central airways with no foreign bodies or aspirated matter, though large amounts of thick mucus plugs were removed through suctioning of the left lower lobe bronchus. A chest CT after 24 h showed the reappearance of total atelectasis of the left lower lobe. Sputum and bronchoalveolar lavage cultures were negative.

A nasogastric tube was inserted on hospital day 2 for continuous gastric decompression. Approximately 1,200 mL of air with gastric contents was removed, and abdominal distension was temporarily relieved. Accumulating gas was observed over the next 48 h, especially during brief clamping experiments, supporting the continued presence of aerophagia as opposed than mere gastric stasis. On hospital day 3, BiPAP was started to enhance ventilation and improve the atelectatic lung. The procedure was stopped after 2 h because of increased abdominal distension, indicative of gastric insufflation with glottic incompetence.

The diagnosis should be differentiated from pneumonia, aspiration pneumonitis, pulmonary embolism, and mucus plug-related atelectasis. Acute colonic pseudo-obstruction was considered. However, it was ruled out due to the presence of severe gastric distension, recurrent episodes in spite of active nasogastric decompression, and a correlation to glottic insufficiency over time. A diagnosis of combined left PNI and RLN injury with refractory aerophagia and recurrent atelectasis was confirmed.

**Figure 2:**
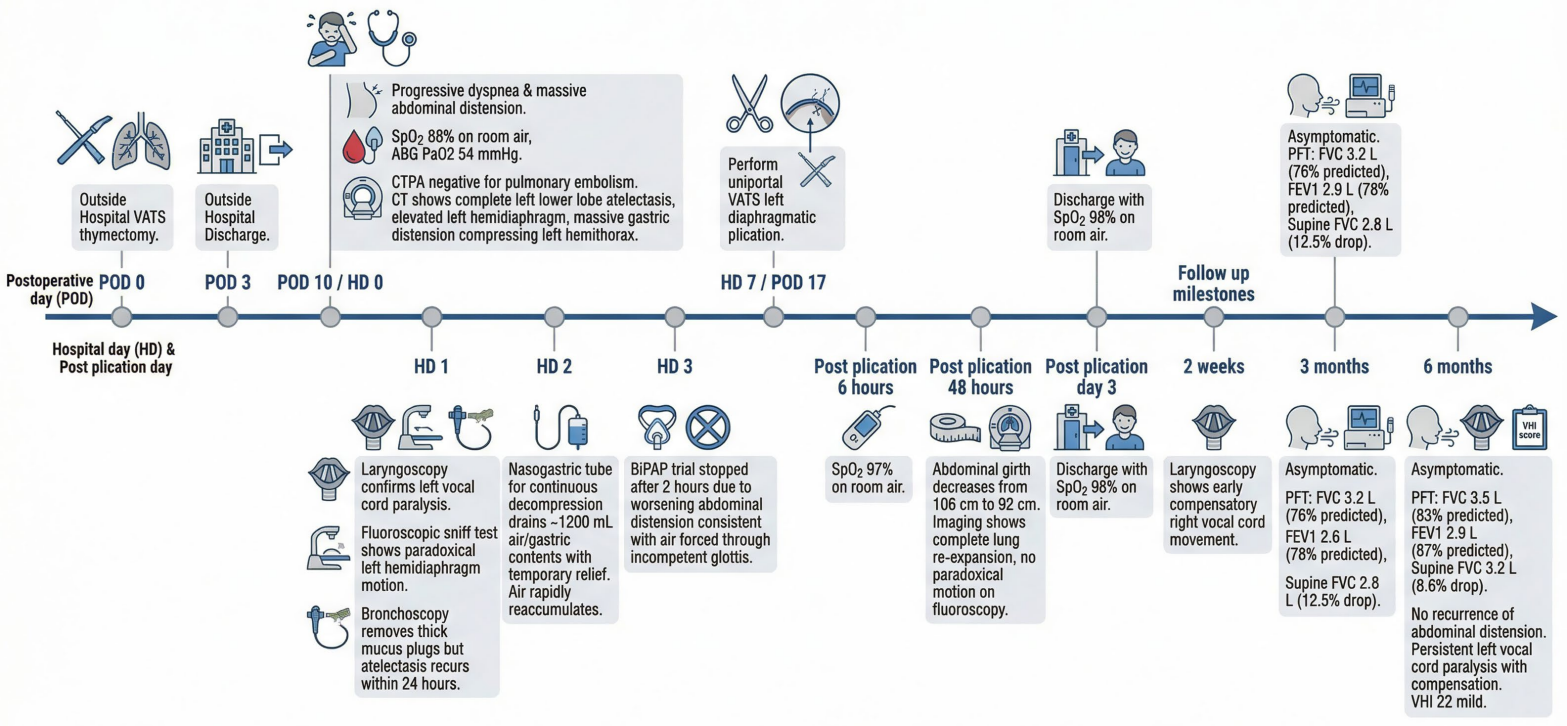
Clinical timeline. Postoperative day 0 indicates VATS thymectomy at an outside hospital. Postoperative day 3, discharge from the outside hospital. Postoperative day 10 presentation to our emergency department with hypoxemic respiratory failure and marked abdominal distension. Hospital day 1, laryngoscopy confirmed left vocal cord paralysis; fluoroscopic sniff testing confirmed paradoxical left hemidiaphragm motion; bronchoscopy removed mucus plugs. Hospital day 2, nasogastric decompression drained approximately 1,200 mL of air and gastric contents with transient improvement. Hospital day 3, BiPAP trial discontinued after 2 h due to worsening abdominal distension. Hospital day 7, corresponding to postoperative day 17, single-incision VATS left diaphragmatic plication performed. Within 6 h, oxygenation normalized in room air. Within 48 h, abdominal girth decreased from 106 cm to 92 cm, and imaging confirmed lung re-expansion. Post-aplication day 3 discharge. Follow-up at 3 and 6 months demonstrated sustained symptom resolution, improved upright and supine spirometry, and persistent but compensated left vocal cord paralysis.

On hospital day 7, we concluded that the patient had not responded to conservative treatment. More than 4 liters per min of supplemental oxygen were required to maintain SpO_2_ above 90%. A decision was made to proceed with surgical intervention at our hospital. The main events and their timings are summarized in Figure 2.

A single-incision VATS approach (3), with all instruments and the camera placed through a single incision, was implemented (4). A 3-cm incision was performed in the sixth intercostal space along the anterior axillary line with a wound protector. A 30-degree 10-mm thoracoscope was used for visualization. Left diaphragmatic plication was performed using a running U-stitch technique as described by Dunning et al. (5). Two rows of continuous non-absorbable #1 polypropylene sutures were placed from anterior to posterior in a U-shaped configuration, incorporating the entire length of the redundant diaphragm to reduce surface area and flatten the dome. Each suture line ran parallel to the phrenic nerve course to avoid further nerve injury, creating approximately 2–3 cm of tissue plication with each pass. The anterior row was placed first, effectively lowering the left hemidiaphragm by approximately 4–5 cm in vertical height (6). Because the intervention was performed 17 days after the thymectomy, the diaphragm retained sufficient thickness without significant atrophy. No mesh or pledgets were required given the adequate tissue quality. The procedure lasted 55 min with an estimated blood loss of less than 10 mL.

The patient’s clinical picture improved rapidly. SpO_2_ increased from 90% on 4 liters per min oxygen to 97% on room air within 6 h of the procedure. Arterial blood gas analysis on postoperative day 1 revealed pH 7.42, PaCO_2_ 38 mmHg, and PaO_2_ 92 mmHg in room air. Dyspnea intensity decreased, and orthopnea resolved. The patient was able to lie flat and supine. His cough resolved, and he was able to clear secretions. Additional bronchoscopy was not required. Abdominal girth decreased from 106 cm to 92 cm within 48 h of the procedure. Stable plication without paradoxical movement was observed on postoperative day 2 by fluoroscopy. Follow-up chest CT scan revealed that the left lower lobe had completely re-expanded and the diaphragm regained normal contour (Figure 1C). The patient was discharged with SpO2 98% on room air on postoperative day 3.

The patient still suffered from left vocal cord paralysis until discharge. Laryngoscopy 2 weeks after discharge revealed initial compensatory motion of the right vocal cord in the midline direction. Speech therapy was recommended. There was no abdominal distension after therapy, and aerophagia gradually resolved. However, vocal cord paralysis persisted. The Voice Handicap Index score was 22, indicating mild voice-related impairment. Maximum inspiratory pressure, maximum expiratory pressure, and total lung capacity were not measured.

## Discussion

Combined PNI and RLN injury is uncommon after cardiothoracic surgery, and it may lead to clinically significant respiratory complications. Tewari (7) reported left PNI and RLN injury with varying degrees of severity after coronary artery bypass grafting. This clinical condition can also be found in other settings (8). Our case adds to the limited literature on combined PNI and RLN injury. This study reported a case of severe aerophagia and gastric distension linked to refractory atelectasis and hypoxemic respiratory failure, and recorded the clinical picture after early diaphragmatic plication.

Cough has three successive phases (9): inspiration, closure of the glottis, and forced expiration. PNI impairs inspiratory capacity by causing diaphragmatic paralysis accompanied by paradoxical motion and diminished vital capacity. RLN injury leads to glottic insufficiency, which prevents the compressive phase required to create high intrathoracic pressure and, in turn, decreases the effectiveness of coughing and airway clearance (10–12). The patient reported here still had recurrent mucus retention and atelectasis following bronchoscopic clearance, which is a typical manifestation of impaired clearance mechanisms. Additionally, as glottic insufficiency persisted, the risk of microaspiration cannot be ruled out. Although no gross aspirated material was observed during bronchoscopy, microaspiration of pharyngeal secretions and gastric refluxate, together with the patient’s inability to effectively clear these secretions, may have played a significant role in contributing to the recurrent atelectasis of the left lower lobe.

Extreme gastric dilatation is one of the most significant symptoms in this case. We hypothesized that ineffective coughing, repeated atelectasis, and the consequent breathing difficulties led to a pattern of tachypnea and open mouth breathing, resulting in aerophagia in the presence of glottic incompetence. This hypothesis is supported by previous reports in which severe respiratory distress was associated with aerophagia, particularly in the context of compromised upper airway protective mechanisms. Under respiratory distress, excessive negative intrathoracic pressure during struggling inspiration can overcome the lower esophageal sphincter, inevitably drawing air into the esophagus and stomach (13–15). Progressive gastric distension likely worsens respiratory mechanics by elevating the hemidiaphragm and compressing the left lower lobe, creating a self-sustaining cycle. As data on video-fluoroscopic swallowing evaluation, esophageal impedance monitoring, and objective peak cough flow measurements are not available, further validation is required.

The mechanism of RLN injury in this case remains uncertain. Several potential etiologies warrant consideration. The thermal effect caused by ultrasonics has been recognized as a risk factor for RLN injury, particularly during dissection near the aortopulmonary window (16, 17). Other mechanisms, including mechanical traction during mediastinal tissue mobilization, direct compression or manipulation of the nerve during thymic dissection, or endotracheal tube cuff-related pressure injury, cannot be excluded (18). Due to the lack of intraoperative video documentation, the mechanism underlying the RLN injury in this case cannot be precisely ascertained.

Several issues regarding surgical experience and technique deserve comment. We cannot definitively determine the operating surgeon’s experience without knowledge of their training background and the number of cases they have performed. In this report, an extended mediastinal dissection of a 1.6-cm mass was performed, which may warrant reflection on the appropriateness of the surgical indication and extent of resection. As reported, masses attached to the phrenic nerve require sharp dissection. Given the occurrence of combined nerve injury following dissection, a more conservative approach or an alternative diagnostic strategy, such as PET-CT or image-guided biopsy, should be considered preoperatively. Additionally, the omission of immediate postoperative laryngoscopy despite recorded hoarseness, combined with the decision to discharge on day 3 without adequate assessment, may reflect a gap in postoperative management protocols. We wish to emphasize several important learning points from this case: (1) surgical intervention should be performed cautiously in carefully selected cases, particularly for small masses without definitive preoperative tissue diagnosis; (2) meticulous techniques should be applied for dissection of masses in close proximity to neurovascular structures, with consideration of nerve monitoring or preservation strategies; (3) comprehensive evaluation of new neurological symptoms should be arranged before hospital discharge; and (4) appropriate follow-up protocols should be formulated for early detection of complications (19, 20). It is also worth noting that even experienced surgeons may encounter unexpected anatomical variations or tumor adherence that increase complication risk, and iatrogenic nerve injury can occur despite optimal technique.

BiPAP may lead to gastric insufflation and increased abdominal distention, especially in the setting of poor upper airway competence (14). In the present case, the patient suffered from increased abdominal distension after BiPAP, and BiPAP was then stopped immediately. This suggests that BiPAP may be a major cause of increased abdominal distension.

Diaphragmatic plication (21) is always performed early in the context of manifest failure of conservative treatment, including a constant need for supplemental oxygen, recurrent atelectasis immediately after bronchoscopic clearing, uncontrollable aerophagia even with nasogastric decompression, and BiPAP intolerance (22–25). In the present case, diaphragmatic plication was implemented 17 days after initial surgery, given his rapid clinical deterioration, failure of multiple conservative interventions, and persistent severe hypoxemia. It is advised to wait 6–12 months after surgery to allow for potential spontaneous nerve recovery (26). We believe that it is reasonable to offer early intervention in cases of life-threatening respiratory failure that are refractory to conservative management. Consistently, recent literature advocates selective early plication in symptomatic patients (27).

A possible disadvantage of plication is that it can prevent nerve regeneration and diaphragmatic excursion in the event of phrenic nerve recovery (28). However, spontaneous recovery of function after complete paralysis of the phrenic nerve is extremely rare, and the extent of recovery closely correlates with the mechanism and severity of the injury (29). Notably, the likelihood of functional recovery can be much lower in cases of iatrogenic injury developed during thoracic surgery. The patient reported here had severe respiratory failure and failed to respond to conservative treatment. Thus, the decision to implement early intervention is reasonable. Additionally, early timing can allow for better diaphragm tissue quality with improved durability of sutures.

There are several key strategies to prevent combined nerve injuries: (1) apply meticulous surgical techniques with careful identification and preservation of the phrenic nerve throughout the procedure; (2) use energy devices wisely and raise awareness about thermal effects to keep a safe distance from neural structures (30); (3) take intraoperative nerve monitoring into account, whose routine use in VATS thymectomy remains debated (31); and (4) perform appropriate patient selection and careful risk–benefit analysis for small or borderline masses; (5) undergo a comprehensive preoperative consultation regarding potential neurological complications. The establishment of institutional protocols for the assessment of voice and diaphragm function in patients after mediastinal surgery may facilitate early detection and intervention (32).

The report has some limitations. First, this was a single-case study. Second, our study failed to objectively assess cough strength and quantify air swallowing. Third, baseline preoperative pulmonary function tests were not performed. Fourth, the analysis of the study depended on external surgical records without intraoperative image validation. Fifth, the follow-up period was relatively short. Long-term follow-up is required to assess possible phrenic nerve recovery and long-term stability of the plication.

## Conclusion

Combined PNI and RLN injury after VATS thymectomy is uncommon but can cause serious respiratory impairment. In this case, ineffective coughing, recurrent atelectasis, and aerophagia with significant gastric distension seemed to constitute a self-perpetuating pathophysiological cycle, which was exacerbated by non-invasive positive pressure ventilation. Single-incision VATS diaphragmatic plication using the running U-stitch technique resulted in rapid recovery of oxygenation, with long-term lung re-expansion and resolution of abdominal distention. However, vocal cord paralysis persisted. To conclude, combined nerve injuries may be considered a differential diagnosis in patients who develop hoarseness and ipsilateral hemidiaphragm elevation after thymectomy. Vigilance is suggested in patients undergoing inter-hospital transfer. Based on this single case observation, early plication may serve as a beneficial therapeutic option upon failure of conservative treatment, especially in the setting of persistent hypoxemia and recurrent atelectasis. Future studies should focus on defining optimal timing of surgical intervention and identifying patient-specific factors that predict the failure of conservative management.

## Data Availability

The original contributions presented in the study are included in the article/supplementary material, further inquiries can be directed to the corresponding author.
